# The impact of obesity-associated glycine deficiency on the elimination of endogenous and exogenous metabolites via the glycine conjugation pathway

**DOI:** 10.3389/fendo.2024.1343738

**Published:** 2024-04-03

**Authors:** Hong Chang Tan, Jean W. Hsu, E Shyong Tai, Shaji Chacko, Jean-Paul Kovalik, Farook Jahoor

**Affiliations:** ^1^Department of Endocrinology, Singapore General Hospital, Singapore, Singapore; ^2^Children’s Nutrition Research Center, Agricultural Research Service, U.S. Department of Agriculture, and Department of Pediatrics, Baylor College of Medicine, Houston, TX, United States; ^3^Department of Medicine, Yong Loo Lin School of Medicine, National University Health System, Singapore, Singapore; ^4^Cardiovascular and Metabolic Disorders Program, Duke-NUS Medical School, Singapore, Singapore

**Keywords:** glycine deficiency, class III obesity, glycine conjugation, acylglycine, bariatric surgery

## Abstract

**Background:**

Glycine is an integral component of the human detoxification system as it reacts with potentially toxic exogenous and endogenously produced compounds and metabolites via the glycine conjugation pathway for urinary excretion. Because individuals with obesity have reduced glycine availability, this detoxification pathway may be compromised. However, it should be restored after bariatric surgery because of increased glycine production.

**Objective:**

To examine the impact of obesity-associated glycine deficiency on the glycine conjugation pathway. We hypothesize that the synthesis rates of acylglycines from endogenous and exogenous sources are significantly reduced in individuals with obesity but increase after bariatric surgery.

**Methods:**

We recruited 21 participants with class III obesity and 21 with healthy weight as controls. At baseline, [1,2-^13^C_2_] glycine was infused to study the glycine conjugation pathway by quantifying the synthesis rates of several acylglycines. The same measurements were repeated in participants with obesity six months after bariatric surgery. Data are presented as mean ± standard deviation, and *p*-value< 0.05 is considered statistically significant.

**Results:**

Baseline data of 20 participants with obesity were first compared to controls. Participants with obesity were significantly heavier than controls (mean BMI 40.5 ± 7.1 vs. 20.8 ± 2.1 kg/m^2^). They had significantly lower plasma glycine concentration (168 ± 30 vs. 209 ± 50 μmol/L) and slower absolute synthesis rates of acetylglycine, isobutyrylglycine, tigylglycine, isovalerylglycine, and hexanoylglycine. Pre- and post-surgery data were available for 16 participants with obesity. Post-surgery BMI decreased from 40.9 ± 7.3 to 31.6 ± 6.0 kg/m^2^. Plasma glycine concentration increased from 164 ± 26 to 212 ± 38 μmol/L) and was associated with significantly higher rates of excretion of acetylglycine, isobutyrylglycine, tigylglycine, isovalerylglycine, and hexanoylglycine. Benzoic acid (a xenobiotic dicarboxylic acid) is excreted as benzoylglycine; its synthesis rate was significantly slower in participants with obesity but increased after bariatric surgery.

**Conclusion:**

Obesity-associated glycine deficiency impairs the human body’s ability to eliminate endogenous and exogenous metabolites/compounds via the glycine conjugation pathway. This impairment is ameliorated when glycine supply is restored after bariatric surgery. These findings imply that dietary glycine supplementation could treat obesity-associated metabolic complications due to the accumulation of intramitochondrial toxic metabolites.

**Clinical trial registration:**

https://clinicaltrials.gov/study/NCT04660513, identifier NCT04660513.

## 1 Introduction

Obesity leads to systemic derangement in the metabolism of various nutrients, including amino acids. In individuals with obesity, plasma concentrations of several amino acids, such as the branched-chain acids (BCAAs), phenylalanine, and tyrosine, are consistently elevated. By contrast, the plasma concentration of glycine is significantly reduced (1–6). Glycine is traditionally categorized as a nutritionally non-essential amino acid, meaning the human body’s glycine requirement can be met through food intake plus endogenous synthesis from other precursors. Yet, in obesity, a state of nutrient oversupply, the supply of this amino acid seems to be deficient. The reason for obesity-associated glycine deficiency was previously unknown until we reported that it is due to impaired *de novo* glycine synthesis secondary to insulin resistance (6). Interestingly, *de novo* glycine synthesis rate increased, and glycine deficiency was corrected in individuals with class III obesity following weight loss after bariatric surgery (6).

Glycine is central to human metabolism and is required in large amounts by the human body. Glycine serves as a substrate to support the synthesis of proteins and is the precursor of several critical biomolecules such as purine, creatine, porphyrin, and glutathione. It is also a primary donor of 1-carbon, essential for various methylation, redox, and biosynthetic reactions (7, 8). In addition, glycine is an integral component of the human detoxification system via the glycine conjugation pathway (7–9). The glycine conjugation pathway is a phase II detoxification system in the liver and is catalyzed by the enzyme *glycine N-acyltransferase*. It involves the transfer of acyl groups from acylCoA (from endogenously or exogenously derived organic acids) in the mitochondria to glycine to form their respective acylglyicine conjugates for urinary excretion (7–9).

The importance of glycine conjugation as an alternative route for the elimination of toxic metabolites is exemplified in patients with inborn errors of metabolism (IEM), who accumulate a large amount of intramitochondrial acylCoAs due to defects in fatty acid and/or amino acid oxidation (10). Similarly, in obesity, the accelerated turnover rates of substrates result in an increased influx of substrates into the mitochondria for oxidation and/or clearance. However, mitochondrial capacity may not increase in tandem. When the influx of substrates exceeds mitochondrial oxidative capacity, these intermediates accumulate as intramitochondrial acylCoAs (11, 12), and the glycine conjugation pathway may be activated as an alternative detoxification pathway (7–9). Since glycine availability is rate limiting for the glycine conjugation reaction (9, 13) and glycine is deficient in patients with obesity, the glycine conjugation pathway may be impaired.

## 2 Aim and hypothesis

Our understanding of the impact of obesity-associated glycine deficiency on the glycine conjugation pathway is limited. Furthermore, it is unclear whether glycine deficiency in individuals with obesity affects the elimination of both endogenous and exogenous metabolites. Our aim in this study is to test the hypothesis that compared to healthy weight individuals, acylglycine synthesis rates are significantly reduced in individuals with class III obesity but will be restored six months after bariatric surgery. Further, obesity-associated glycine deficiency affects the elimination of both endogenous and exogenous metabolites. To achieve our aim, we utilized stable-isotope tracer methods to study the glycine conjugation pathway by quantifying the synthesis and excretion rates of several endogenously and exogenously derived acylglycines in participants with class III obesity and healthy weight. We then repeated the measurements in the same individuals with class III obesity six months after bariatric surgery.

## 3 Methods

### 3.1 Subjects

This study was approved by the SingHealth Centralized Institutional Review Board (CIRB Ref: 2018/2714), and all participants provided written informed consent. We recruited 21 participants with class III obesity and 21 with normal weight. Participants with class III obesity were recruited from patients attending the Singapore General Hospital’s obesity clinic who were scheduled for bariatric surgery. They were recruited if they were between 21 and 65 years old and had a BMI ≥ 32.5 kg/m^2^ with obesity-related complications. They were excluded if they were receiving insulin treatment, consumed excessive alcohol (> 1 drink/day for females or > 2 drinks/day for males), received systemic corticosteroid treatment, or had existing cardiovascular, kidney, or liver disorders. Individuals with a healthy weight (BMI< 25 kg/m^2^) were recruited from our healthy volunteer database. These participants were excluded if they had diabetes mellitus or any significant chronic illness.

All eligible participants underwent stable-isotope tracer studies at baseline. Participants with obesity then underwent bariatric surgery and returned six months later for the same stable-isotope studies to measure the post-surgery changes from baseline.

### 3.2 Stable-isotope infusion

[1,2-^13^C_2_] glycine (99 atom% ^13^C) was used to study the glycine conjugation pathway by quantifying the synthesis rates of several acylglycines. The tracer was purchased as a sterile and pyrogen-free compound (Cambridge Isotope Laboratories, MA) and reconstituted within 24 hours of the intravenous infusion.

Participants were admitted to the SingHealth Investigational Medical Unit one day before the stable-isotope infusion. They were asked to refrain from smoking, drinking coffee and alcohol, and vigorous exercise (more than 1 hour of high-intensity physical activity) during the 24 hours before the study. Dinner was prepared by the hospital’s kitchen, with the calories kept similar to their habitual intake. Meal energy composition consisted of 55% from carbohydrates, 33% from fat %, and 15% from protein. Both groups’ total daily protein intake, hence dietary glycine, was similar. Subjects also drank 200 mL of zero-calorie soda containing benzoic acid and fasted from 10:00 PM onwards.

After an 8-hour overnight fast, two intravenous cannulas were inserted into opposite arms; one for the infusion of tracers and the other for blood draws. A hand warmer was used to arterialize the venous blood collected. After collecting baseline blood and urine samples for metabolite analysis and background isotopic enrichments (IEs), a primed-constant infusion of [1,2-^13^C_2_] glycine (Prime=8 µmol·kgFFM^-1^, Infusion=8 µmol·kgFFM^-1^·h^-1^) was started and maintained for the next 7 hours. Blood samples were collected hourly from the 4^th^ to 6^th^ hour of infusion and every 15 minutes during the last hour. Urine samples were collected at baseline (from 10 PM until the start of the infusion) and at 2, 4, 5, 6, and 7 hours.

### 3.3 Laboratory analyses

#### 3.3.1 Plasma amino acid and acylcarnitine profiling

Plasma amino acid (AA) concentrations were measured by ultra-high performance liquid chromatography (ACQUITY H-Class System, Waters Corporation, MA, USA) using pre-column derivatization with 6-aminoquinolyl-N-hydroxysuccinimidyl carbamate (Waters AccQ×Tag™ assay kit, MA, USA) and norvaline (Sigma Aldrich, MO, USA) as internal standard. Plasma samples were deproteinized with 10% sulfosalicylic acid dihydrate and derivatized using AccQ-Fluor ™ derivative reagent. The derivatized AAs were separated using gradient-based ACQUITY UPLC BEH C18 column (130 Å, 1.7 μM, 2.1 mm x 150 mm) with ACQUIT UPLC Tunable UV (TUV) detector (6). Total plasma cysteine (cystine plus cysteine) concentration was measured by *in vitro* isotope dilution as described previously (14). A known quantity of U-^13^C_3_-cysteine (Cambridge Isotope Laboratories Inc.) was added as an internal standard to the baseline plasma samples; dithiothreitol (60 mmol/L in 0.1 mol sodium tetraborate/L) was added to convert cystine to cysteine Cysteine was then alkylated by adding iodoacetamide (0.5 mol/L) in 0.1 mol ammonium bicarbonate/L. Alkylated cysteine was converted into its DANS [5-(dimethylamino)-1-napthalene sulfonamide] derivative and analyzed by liquid chromatography–mass spectrometry. The ions were then analyzed by SRM mode. The transitions observed were: m/z 412 to 170 & 415 to 170.

Acylcarnitines are formed by the conjugation of acylCoAs with free carnitine in the mitochondria and exported back into the plasma. Therefore, the measurement of acylcarnitines in the plasma pool reflects the intramitochondrial content of their respective acylCoAs. In addition, plasma concentrations of acylcarnitines of different chain lengths correspond to their respective acylCoAs. Short-chain acylcarnitines (C3-C5) are derived from the metabolism of amino acids such as the BCAAs. In contrast, medium-chain acylcarnitines (C6-C12) and long-chain acylcarnitines (C14-C22) reflect oxidation of medium and long-chain fatty acids, respectively. Acetylcarnitine (C2) represents intra-mitochondrial acetylCoA, the final common metabolic product of substrate oxidation.

In this study we measured the plasma concentrations of acetylcarnitine (C2), propionylcarnitine (C3), butyrylcarnitine (C4), and isovalerylcarnitine (C5), octanoylcarnitine (C8), myristoylcarnitine (C14) and palmitoylcarnitine (C16) by *in vivo* isotope dilution using internal standards from labelled carnitine standards set B (NSK-B, Cambridge Isotope Laboratories Inc.) and analyzed by LC tandem MS (TSQ Altis; Thermo Scientific) as previously described (5). The ions were then analyzed by SRM mode, and the transitions observed were: Acetylcarnitine m/z 204 to 85 & 207 to 85; Propionylcarnitine m/z 218 to 85 & 221 to 85; Butyrylcarnitine m/z 232 to 85 & 235 to 85; Isovalerylcarnitine m/z 246 to 85 & 255 to 85; Octanoylcarnitine m/z 288 to 85 & 291 to 85; Myristoylcarnitine m/z 372 to 85 & 381 to 85; Palmitoylcarnitine m/z 400 to 85 & 403 to 85.

#### 3.3.2 Acylglycine profiling

Urine collected from 10:00 PM until the end of the study was pooled and used to measure concentrations of various acylglycines. Acetylglycine is derived from acetylCoA, while isobutyrylglycine, tigylglycine, and isovalerylglycine are formed from glycine conjugation with their BCAA-derived acylCoAs. Hexanoylglycine and octanoylglycine are synthesized from medium-chain fatty acid acylCoA. To estimate the ability of the glycine conjugating pathway to eliminate exogenously derived compounds, we measured bezoylglycine (hippuric acid), which is formed by the conjugation of benzoic acid (consumed from the diet soda of the evening meal) with glycine. The internal standards (^2^H_5_-acetylglycine, ^2^H_2_-isobutyrylglycine, ^13^C_2_,^15^N-tigylglycine, ^13^C_2_,^15^N- isovalerylglycine, ^13^C_2_,^15^N- hexanoylglycine, ^2^H_2_-octanoylglycine, ^2^H_2_- bezoylglycine) used were from Cambridge Isotope Laboratories Inc. The urinary acylglycine was butylated according to the method of Hobert et al. (15) & Fisher et al. (16) and analyzed by LC tandem MS (TSQ Altis, Thermo Scientific). The ions were then analyzed by SRM mode. The transitions observed were: Acetylglycine m/z 174 to 76 & 179 to 79; Isobutyrylglycine m/z 202 to 76 & 204 to 78; Tigylglycine m/z 214 to 83 & 217 to 83; Isovalerylglycine m/z 216 to 76 & 219 to 79; Hexanoylglycine m/z 230 to 76 & 233 to 79; Octanoylglycine m/z 258 to 76 & 260 to 78; and Benzoylglycine m/z 236 to 105 & 241 to 110. Urine acylglycine concentrations were corrected for renal function and expressed as mmol/mol creatinine (crt).

#### 3.3.3 Isotopic enrichments

Intracellular glycine isotopic enrichment was measured in red blood cells (RBC) using liquid chromatography-tandem mass spectroscopy (LC-MS/MS). Briefly, RBC free glycine was converted into its DANS [5-(dimethylamino)-1-napthalene sulfonamide] derivative and analyzed using a Kinetex C18 2.6μ 100 × 2.1 mm column (Phenomenex, Torrance, CA) on a triple quadrupole mass spectrometer (TSQ Altis; Thermo Scientific, San Jose, CA). The ions were then analyzed by SRM (selected reaction monitoring) mode. The transitions observed were precursor ions m/z 309, and 311 to product ion m/z 170.

IEs of various acylglycines in urine were measured using liquid chromatography-tandem mass spectroscopy (LC-MS/MS). Acylglycine was butylated and analyzed using an Omega C18 2.6μ 100 × 2.1 mm column (Phenomenex, Torrance, CA) on a triple quadrupole mass spectrometer (TSQ Altis; Thermo Scientific, San Jose, CA). The ions were then analyzed by SRM mode. The transitions observed were: Acetylglycine m/z 174 to 76 & 176 to 78; Isobutyrylglycine m/z 202 to 76 & 204 to 78; Benzoylglycine m/z 236 to 105 & 238 to 105; Tigylglycine m/z 214 to 83 & 216 to 83; Isovalerylglycine m/z 216 to 76 & 218 to 78; and Hexanoylglycine m/z 230 to 76 & 232 to 78; Octanoylglycine m/z 258 to 76 & 260 to 78; and Benzoylglycine m/z 236 to 105 & 238 to 105.

#### 3.3.4 Acylglycine kinetics

The kinetics of octanoylglycine was excluded because its urinary isotopic enrichments were too low to be measured reliably.

The fractional (FRS) and absolute (ASR) synthesis rates of acetylglycine, isobutyrylglycine, tigylglycine, isovalerylglycine, octanoylglycine, hexanoylglycine, and benzoylglycine were estimated according to the precursor-product equations:


$$\text{FS}{\text{R}}_{\text{acylglycine}}\left(\%\;\text{pool}/\text{d}\right)\;=\Delta \text{I}{\text{E}}_{\text{acylglycine}}/\;\text{I}{\text{E}}_{\text{RBC}}\text{x}\;24\;\left(\text{hr}\right)$$

Where Δ IE_acylglycine_ = isotopic enrichment slope of acylglycine (%/h) based on the M+2 enrichment of acyglycine from time 0 to 7 hour. IE_RBC_ = m+2 enrichment of RBC free glycine (used as a proxy for intracellular glycine enrichment) during the final hour of infusion.

The ASR of acylglycines were calculated as described below and expressed as mmol/mol crt/day:


$$\text{AS}{\text{R}}_{\text{acylglycine}\;}\left(\;\text{mmol}/\text{mol}\;\text{crt}/\text{day}\right)=\;\text{FS}{\text{R}}_{\text{acylglycine}}\text{x}\;\text{urine}\;\text{acylglycine}\;\text{concentration}$$

### 3.4 Statistics

Our previous study indicated that bariatric surgery increases plasma glycine concentration by 26 mmol/L (standard deviation = 26) (6, 17, 18). We hypothesized that this increase will result in a significant improvement in acylglycine synthesis rate, hence, we will need to recruit 21 subjects with class III obesity for a power of more than 80% at a 0.05 significance level. Therefore, 21 normal-weight participants were recruited as controls to compare the differences in acylglycine kinetics at baseline. The distribution of continuous data was first examined, and data with a normal distribution were presented as mean ± standard deviation. Data that did not follow a normal distribution was presented as medians with interquartile range. Statistical differences in metabolic parameters between participants with class III obesity and healthy weight were sought using the unpaired t-test. If the residuals did not follow a normal distribution, the Mann–Whitney U test was used.

Similarly, significant changes in metabolic parameters after bariatric surgery were tested using the paired *t*-test. Wilcoxon’s Signed Rank test was used if the residuals did not follow a normal distribution. To examine the relationship between glycine availability and glycine conjugation pathway, linear regression was performed with benzoylglycine ASR as the dependent variable and plasma glycine concentration as the independent variable. P values< 0.05 were considered statistically significant. Statistical testing was performed using STATA version 17 (Stata Corp) and Prism version 9 (GraphPad Software Inc.).

## 4 Results

### 4.1 Baseline characteristics, plasma amino acid concentration, and plasma acylcarnitines

One healthy weight control and one participant with obesity were excluded because their urine samples were not collected due to menstruation. Results from 20 participants with healthy weight and 20 participants with obesity (n = 20) with complete data were analyzed. Participants with healthy weight and obesity were not different in age (40 ± 10 vs. 41 ± 9 years, p = 0.726), and there was an equal number of females (n = 16 in each group). Participants with obesity were significantly heavier than controls, with a body weight of 104.9 ± 15.6 vs. 56.4 ± 8.8 kg, p< 0.0001, and body mass index of 40.5 ± 7.1 vs. 20.8 ± 2.1 kg/m^2^, p< 0.0001.

Compared to healthy weight controls, participants with obesity had significantly lower plasma concentrations of glycine and serine (**Table 1**). By contrast, plasma concentrations of several nutritionally non-essential amino acids (cysteine, tyrosine, alanine, aspartate, and glutamate) and nutritionally essential amino acids (leucine, isoleucine, valine, methionine, phenylalanine, and lysine) were significantly higher in those with obesity (**Table 1**).

**Table 1:** Plasma amino acid concentration of participants with healthy weight and class III obesity.

(µmol/L)	Healthy weight (n = 20)	Class III Obesity (n = 20)	*P* value
Nutritionally non-essential amino acids			
Glycine	209 ± 50	168 ± 30	0.0034
Serine	122 ± 14	105 ± 18	0.0023
Glutamine	452 ± 74	471 ± 56	0.3546
Total Cysteine	272 ± 39	338 ± 49	<0.0001
Tyrosine	50 ± 10	66 ± 8	<0.0001
Arginine	77 (66-89)	78 (72-97)	0.6017
Proline	131 (103-164)	149 (128-173)	0.2315
Alanine	227 (189-269)	314 (289-341)	<0.0001
Asparagine	35 ± 6	35 ± 6	0.8330
Aspartate	2 ± 1	3 ± 1	0.0030
Glutamate	27 ± 15	54 ± 24	0.0001
Nutritionally essential amino acids			
Leucine	110 ± 17	137 ± 26	0.0004
Isoleucine	52 ± 9.3	68 ± 13	0.0001
Valine	209 ± 34	253 ± 43	0.0008
Methionine	19 (12-22)	21 (20-24)	0.0080
Phenylalanine	56 ± 6	66 ± 8	<0.0001
Threonine	104 ± 25	116 ± 39	0.1733
Lysine	163 ± 32	202 ± 31	0.0004
Histidine	75 ± 9	70 ± 10	0.0642
Tryptophan	39 ± 7	39 ± 6	0.9313

Values are presented as median (inter-quartile range) or mean ± standard deviation. P value< 0.05 is considered as statistically significant.

Participants with class III obesity also had significantly higher plasma concentrations of propionylcarnitine (C3), butyrylcarnitine (C4), isovalerylcarnitine (C5), and myristoylcarnitine (C14) compared to healthy weight controls. However, there were no statistically significant differences in the plasma concentrations of acetylcarnitine (C2), octanoylcarnitine (C8), and palmitoylcarnitine (C16) between the two groups (**Table 2**).

**Table 2:** Plasma acylcarnitine concentration of participants with healthy weight and class III obesity.

(µmol/L)	Healthy weight (n = 20)	Class III Obesity (n = 20)	*P* value
Acetylcarnitine (C2)	13.09 ± 3.63	15.49 ± 6.23	0.1446
Propionylcarnitine (C3)	0.35 ± 0.13	0.45 ± 0.15	0.0376
Butyrylcarnitine (C4)	0.065 (0.051-0.076)	0.126 (0.090-0.213)	<0.0001
Isovalerylcarnitine (C5)	0.056 ± 0.033	0.094 ± 0.051	0.0092
Octanoylcarnitine (C8)	0.107 (0.090-0.162)	0.115 (0.089-0.163)	0.8201
Myristoylcarnitine (C14)	0.020 ± 0.004	0.027 ± 0.009	0.0046
Palmitoylcarnitine (C16)	0.121 ± 0.022	0.135 ± 0.029	0.1115

Values are presented as median (inter-quartile range) or mean ± standard deviation. P value < 0.05 is considered as statistically significant.

### 4.2 Baseline acylglycine concentrations and kinetics

**Figure 1:**
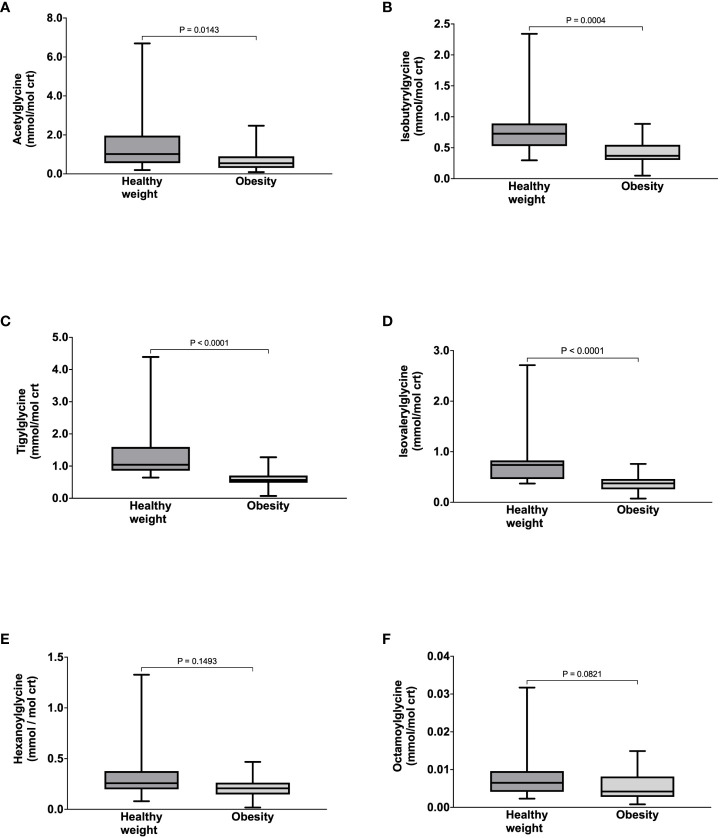
Urine concentrations of Acetyglglycine **(A)**, Isobutyrylglycine **(B)**, Tigylglycine **(C)**, Isovalerylglycine **(D)**, Hexaoylglycine **(E)**, and Octanoylglycine **(F)** in participants with healthy weight and class III obesity.

Compared to healthy weight controls, participants with obesity had significantly lower urine concentrations of acetylglycine [0.54 (0.30-0.90) vs. 1.02 (0.55-1.96) mmol/mol crt, p = 0.0143] (**Figure 1A**), isobutyrylglycine [0.37 (0.30-0.55) vs. 0.73 (0.53-0.89) mmol/mol crt, p = 0.0004] (**Figure 1B**), tigylglycine [0.57 (0.48-0.71) vs. 1.04 (0.86-1.60) mmol/mol crt, p< 0.0001] (**Figure 1C**), and isovalerylglycine [0.37 (0.26-0.46) vs. 0.74 (0.46-0.83) mmol/mol crt, p< 0.0001] (**Figure 1D**). There were no significant between-group differences in the urine concentration of hexanoylglycine [0.32 (0.15-0.26) vs. 0.26 (0.20-0.38) mmol/mol crt, p = 0.1493] (**Figure 1E**) or octanoylglycine [0.32 (0.15-0.26) vs. 0.26 (0.20-0.38) mmol/mol crt, p = 0.0821] (**Figure 1F**). Interestingly, the FSR and ASR of acetylglycine, isobutyrylglycine, tigylglycine, isovalerylglycine, and hexanoylglycine were also significantly slower in participants with obesity than healthy weight controls (**Table 3**).

**Table 3:** Acylglycine kinetics of participants with healthy weight and class III obesity.

	Healthy weight (n = 20)	Class III Obesity (n = 20)	*P* value
Acetylglycine			
FSR (% pool/day)	433 ± 107	316 ± 107	0.0014
ASR (mmol/mol crt/day)	4.70 (2.44-6.13)	1.52 (0.92-3.71)	0.0018
Isobutyrylglycine			
FSR (% pool/day)	448 ± 94	385 ± 102	0.0509
ASR (mmol/mol crt/day)	3.46 (2.26-4.11)	1.49 (0.99-1.49)	<0.0001
Tigylglycine			
FSR (% pool/day)	565 ± 127	443 ± 89	0.0012
ASR (mmol/mol crt/day)	6.96 (4.26-9.09)	2.35 (1.99-3.76)	<0.0001
Isovalerylglycine			
FSR (% pool/day)	606 ± 126	435 ± 77	<0.0001
ASR (mmol/mol crt/day)	4.53 (2.76-5.41)	1.53 (1.27-1.93)	<0.0001
Hexanoylglycine			
FSR (% pool/day)	436 ± 91	340 ± 79	0.0010
ASR (mmol/mol crt/day)	1.19 (0.88-1.51)	0.62 (0.45-1.09)	0.0143

Values are presented as median (inter-quartile range) or mean ± standard deviation. P value< 0.05 is considered statistically significant. FSR, Fractional Synthesis Rate; ASR, Absolute Synthesis Rate; crt, creatinine.

### 4.3 Post-surgery changes in clinical parameters, plasma amino acids, and plasma acylcarnitines

Four participants with obesity were lost to follow-up, and the baseline sample for one individual was not available. Hence, paired results (before and after bariatric surgery) of 16 participants with obesity were analyzed. These participants underwent bariatric surgery (14 sleeve gastrectomy and 2 Roux-en-Y gastric bypass) and returned for their follow-up visit at a median of 6.5 (5.9-8.9) months. They experienced significant reductions in body weight from 106.1 ± 16.5 to 82.0 ± 13.7 kg, p< 0.0001, and BMI from 40.9 ± 7.3 to 31.6 ± 6.0 kg/m^2^, p< 0.0001. Post-surgery plasma glycine and serine concentrations increased significantly. In contrast, the plasma concentrations of the nutritionally non-essential amino acids, tyrosine, proline, aspartate, and glutamate, and nutritionally essential amino acids, isoleucine, valine, methionine, and phenylalanine decreased significantly (**Table 4**).

**Table 4:** Plasma amino acid concentrations of participants with class III obesity before and six months after bariatric surgery.

(µmol/L)	Pre-surgery (n = 16)	Post-surgery (n = 16)	*P* value
Nutritionally non-essential amino acids			
Glycine	164 ± 26	212 ± 38	<0.0001
Serine	103 ± 19	114 ± 21	0.0166
Glutamine	468 ± 51	470 ± 49	0.8338
Total Cysteine	337 ± 48	337 ± 32	0.9713
Tyrosine	66 ± 8	51 ± 5	<0.0001
Arginine	83 ± 18	85 ± 14	0.6178
Proline	144 ± 22	129 ± 18	0.0119
Alanine	312 ± 28	250 ± 34	<0.0001
Asparagine	34 ± 6	36 ± 8	0.4716
Aspartate	3 ± 1	2 ± 1	0.0020
Glutamate	54 ± 26	32 ± 18	0.0005
Nutritionally essential amino acids			
Leucine	134 ± 21	123 ± 16	0.1105
Isoleucine	66 ± 10	56 ± 6	0.0007
Valine	248 ± 38	224 ± 22	0.0262
Methionine	20 (20-23)	19 (17-20)	<0.0001
Phenylalanine	67 ± 8	57 ± 6	0.0001
Threonine	111 ± 30	101 ± 25	0.2628
Lysine	197 ± 30	182 ± 25	0.0783
Histidine	69 ± 8	75 ± 8	0.0779
Tryptophan	40 ± 7	37 ± 3	0.0947

Values are presented as median (inter-quartile range) or mean ± standard deviation. P value< 0.05 is considered as statistically significant.

The plasma concentrations of propionylcarnitine, butyrylcarnitine, and isovalerylcarnintine decreased significantly post-surgery, while plasma acetylcarnitine concentration increased significantly. No statistically significant changes in octanoylcarnitine, myristoylcarnintine, and palmitoylcarnitine were observed (**Table 5**).

**Table 5:** Plasma acylcarnitine concentrations of participants with class III obesity before and six months after bariatric surgery.

µmol/L	Pre-surgery (n = 16)	Post-surgery (n = 16)	*P* value
Acetylcarnitine (C2)	14.83 ± 5.72	17.94 ± 6.83	0.0435
Propionylcarnitine (C3)	0.44 ± 0.14	0.34 ± 0.07	0.0045
Butyrylcarnitine (C4)	0.13 ± 0.05	0.09 ± 0.06	0.0006
Isovalerylcarnitine (C5)	0.09 ± 0.05	0.05 ± 0.02	0.0162
Octanoylcarnitine (C8)	0.13 ± 0.05	0.12 ± 0.04	0.3453
Myristoylcarnitine (C14)	0.03 ± 0.01	0.02 ± 0.01	0.0737
Palmitoylcarnitine (C16)	0.14 ± 0.03	0.14 ± 0.04	0.9584

Values are presented as median (inter-quartile range) or mean ± standard deviation. P value< 0.05 is considered as statistically significant.

### 4.4 Acylglycine concentration and kinetics

**Figure 2:**
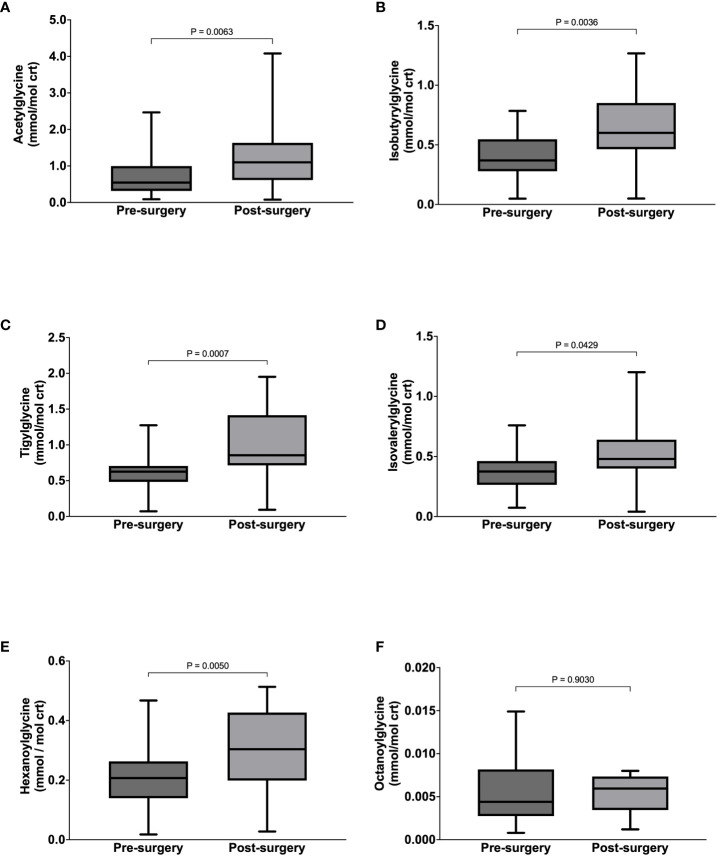
Urine concentrations of Acetyglglycine **(A)**, Isobutyrylglycine **(B)**, Tigylglycine **(C)**, Isovalerylglycine **(D)**, Hexaoylglycine **(E)**, and Octanoylglycine **(F)** in participants with class III obesity before and six months after bariatric surgery.

After bariatric surgery, there were significant increases in the urinary concentrations of acetylglycine from 0.54 (0.32-1.00) to 1.10 (0.61-1.63) mmol/mol crt, p = 0.0063 (**Figure 2A**), isobutyrylglycine from 0.40 ± 0.18 to 0.64 ± 0.31 mmol/mol crt, p = 0.0036 (**Figure 2B**), tigylglycine from 0.62 ± 0.29 to 1.01 ± 0.47 mmol/mol crt (**Figure 2C**), isovalerylglycine from 0.38 ± 0.18 to 0.54 ± 0.29 mmol/mol crt, p = 0.0214 (**Figure 2D**), and hexanoylglycine from 0.22 ± 0.11 to 0.30 ± 0.13 mmol/mol crt, p = 0.0050 (**Figure 2E**). However, urine octanoylglycine concentration was not significantly different from baseline (**Figure 2F**). Acetylglycine ASR increased significantly post-surgery, but the post-surgery increase in its FSR did not reach statistical significance (**Table 6**). On the other hand, FSRs and ASRs of isobutyrylglycine, tigylcine, isovalerylglycine, and hexanoylglycine were significantly higher post-surgery (**Table 6**).

**Table 6:** Acylglycine kinetics of participants with class III obesity before and six months after bariatric surgery.

	Pre-surgery (n = 16)	Post-surgery (n = 16)	*P* value
Acetylglycine			
FSR (% pool/day)	317 ± 112	365 ± 83	0.0775
ASR (mmol/mol crt/day)	1.52 (0.92-1.52)	3.92 (2.29-6.19)	0.0013
Isobutyrylglycine			
FSR (% pool/day)	378 ± 94	448 ± 93	0.0098
ASR (mmol/mol crt/day)	1.48 ± 0.70	2.75 ± 1.14	0.0002
Tigylglycine			
FSR (% pool/day)	442 ± 89	510 ± 80	0.0433
ASR (mmol/mol crt/day)	2.72 ± 1.28	5.18 ± 2.43	0.0002
Isovalerylglycine			
FSR (% pool/day)	429 ± 78	554 ± 97	0.0002
ASR (mmol/mol crt/day)	1.63 ± 0.68	3.00 ± 1.63	0.0016
Hexanoylglycine			
FSR (% pool/day)	342 ± 78	413 ± 62	0.0013
ASR (mmol/mol crt/day)	0.79 ± 0.52	1.25 ± 0.59	0.0004

Values presented as median (inter-quartile range) or mean ± standard deviation. P value< 0.05 is considered as statistical significant. FSR, Fractional Synthesis Rate; ASR, Absolute Synthesis Rate; crt, creatinine.

### 4.5 Benzoylglycine concentration and kinetics

**Figure 3:**
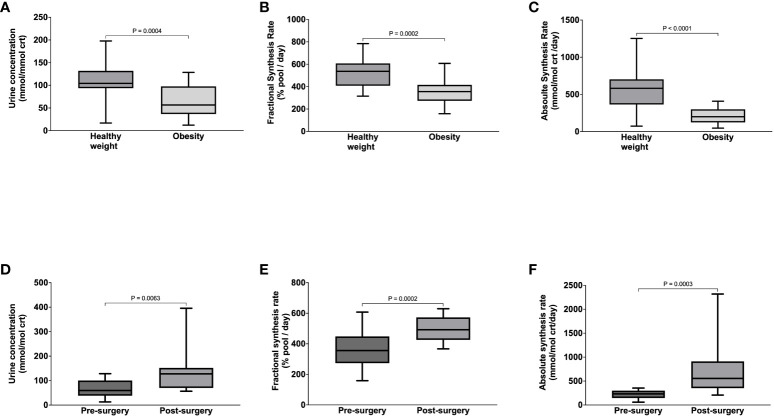
Benzoylglycine urine concentration **(A)**, Fractional Synthesis Rate **(B)**, Absolute Synthesis rate **(C)** in participants with healthy weight and class III obesity, and Benzoylglycine urine concentration **(D)**, Fractional Synthesis Rate **(E)**, and Absolute Synthesis rate **(F)** in participants with class III obesity before and six months after bariatric surgery.

**Figure 4:**
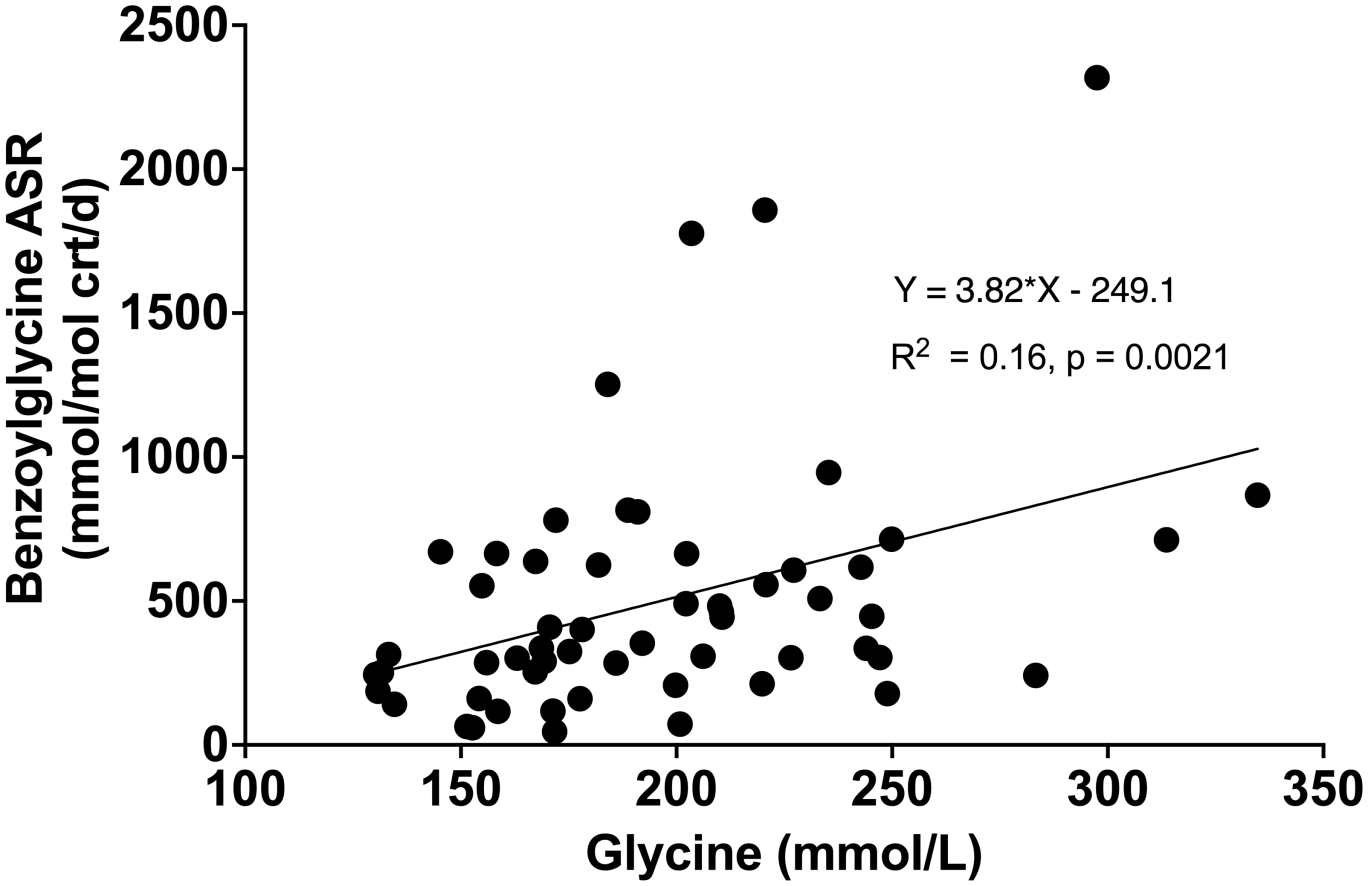
Relationship between plasma glycine concentration with benzoylglycine absolute synthesis rate (ASR).

Compared to healthy weight controls, the urine concentration of benzoylglycine was significantly lower in participants with obesity (63 ± 34 vs. 112 ± 45 mmol/mol crt, p = 0.0004) (**Figure 3A**) due to significantly slower FSR (363 ± 112 vs. 521 ± 128% pool/day, p = 0.0002) (**Figure 3B**) and ASR (211 ± 104 vs. 557 ± 256 mmol/mol crt/day, p < 0.0001) (**Figure 3C**). Following surgery, urine benzoylglycine concentration increased from 59.7 (38.3-100.60) to 127.4 (70.0 -152.2) mmol/mol crt (p = 0.0063) (**Figure 3D**). There were similar significant increases in benzoylglycine FSR (368 ± 121 to 492 ± 83% pool/day) (**Figure 3E**), and ASR [232 (146-299) to 554 (352-912) mmol/mol crt/day, p = 0.0003] (**Figure 3F**). Regression analysis also showed a significant association between plasma glycine concentration and benzoylglycine ASR (model R^2^ = 0.16, Beta coefficient = 3.81, p = 0.0021) (**Figure 4**).

## 5 Discussion

Our study examined the impact of obesity-associated glycine deficiency on the glycine conjugation pathway. Whereas substrate turnover rates are increased in individuals with obesity leading to the accumulation of metabolically toxic intramitochondral aylCoAs, paradoxically plasma glycine concentration is decreased. We found that in patients with class III obesity, the elimination rate of various endogenously produced metabolites and exogenous benzoic acid as acylglycines was impaired. Following bariatric surgery, however, glycine availability improved, and this was associated with increased synthesis and excretion of these acylglycines.

Participants with obesity in our study had higher plasma concentrations of amino acids such as the BCAAs, aromatic amino acids, and alanine compared to healthy weight controls. By contrast, glycine and serine plasma concentrations were significantly lower. These findings are consistent with those reported in earlier studies by us and others (1–6); and such changes in amino acid profile are independent predictors of adverse cardiometabolic outcomes (17, 18). The reasons why the plasma concentration of glycine is reduced while other amino acids, such as the BCAAs, are elevated in individuals with obesity have been elusive. However, we recently showed, using isotopically labelled tracers, that the lower plasma glycine concentration in individuals with obesity can be explained by the slower rate of *de novo* glycine synthesis (6) and elevated BCAAs can be attributed to an accelerated rate of protein breakdown in skeletal muscles due to insulin resistance (4, 5). Glycine and serine are interconvertible via *serine hydroxymethytransferase*, which explains why glycine and serine trend in the same direction (19, 20). Our results also showed significantly higher plasma concentrations of glycine and serine post-surgery, which is due to faster rates of *de novo* glycine synthesis from reduced insulin resistance (6). Similarly, improved insulin-mediated suppression of skeletal muscle breakdown and BCAA oxidation (5, 21, 22) leads to lower post-surgery concentrations of BCAAs (except leucine), aromatic amino acids, methionine, proline, alanine, aspartate, and glutamate. The interaction between BCAA and glycine metabolism has been studied in animal models of obesity, which found that glycine participates in the disposal of excess nitrogen from skeletal muscle BCAA oxidation via the urea cycle, and glycine deficiency may also contribute to higher BCAA levels in obesity. Pharmacologic inhibition of BCAA oxidation increases skeletal muscle and plasma glycine levels and improves insulin resistance (23). However, dietary glycine supplementation lowered BCAAs in the muscle but did not improve insulin resistance. Extrapolating such findings directly to humans is challenging but provides a framework to test additional hypotheses in future studies.

The concentrations of acylcarnitines in plasma reflect the amount and types of acylCoAs in the mitochondria and provide an overview of substrate metabolism at the cellular level. In this study, the higher plasma concentrations of propionylcarnitine (C3), butyrylcarnitine (C4), and isovalerylcarnitine (C5) in individuals with obesity compared to controls indicate the higher turnover rates and oxidation of BCAAs. Similarly, the higher plasma myristoylcarnitine (C14) in participants with obesity indicates accelerated rates of lipolysis and free fatty acid oxidation. Although the higher acetylcarnitine, octanoylcarnitine, and palmitoylcarnitine concentrations in individuals with obesity did not achieve statistical significance, our overall findings are consistent with the notion that obesity is associated with faster substrate turnover rates resulting in the intramitochondrial production and accumulation of various amino acid and lipid oxidation intermediates (11, 12). The reduction of BCAA-derived acylcarnitines: propionylcarnitine (C3), butyrylcarnitine (C4), and isovlearylcarnitine (C5) after bariatric surgery indicates a decrease in BCAA flux from muscle protein breakdown and its oxidation rate following improvement in insulin resistance (3, 5, 21). The lack of statistically significant reductions in medium or long-chain acylcarnitines after surgery indicate that free fatty acid flux and oxidation remained elevated. These findings are expected as post-surgery dietary calorie intake remains low during the first six months after bariatric surgery (5, 9, 21). In this state of negative calorie balance, the body’s energy requirement is dependent on the oxidation of fatty acids mobilized from adipose tissue (24, 25). This interpretation is supported by the higher post-surgery concentration of acetylcarnitine, which reflects the elevated intramitochondrial acetylCoAs generated as the final product of fatty acid beta-oxidation. Ketone bodies generated from acetylCoA can be further utilized by various organs as an energy source. Oxidative stress levels are increased in individuals with obesity due to higher production of reactive oxygen species as a byproduct of accelerated free fatty oxidation. However, this is less of a concern post-surgery despite a reliance on lipid oxidation, as multiple studies have demonstrated a reduction in oxidative stress post-bariatric surgery (26, 27). Weight loss stabilizes 12 months after bariatric surgery (5, 9, 21), and during this phase, the associated changes in nutrient intake will need to be considered for any additional alterations in metabolite levels.

We measured the urine concentrations and synthesis rates of various acylglycines to examine the ability of the glycine conjugation pathway to eliminate potentially toxic endogenously derived metabolites. The decrease in urine isobutyrylglycine and isovalerylglycine have been described in individuals with obesity (5). However, this is the first human study to use stable isotope tracers to confirm the impact of obesity-associated glycine deficiency on the glycine conjugation pathway. We found that at baseline, urine concentrations of acetylglycline and BCAA-derived acylglycines (isobutyrylglycine, tigylglycine, and isovalerylglycine) were significantly lower in individuals with obesity compared to healthy weight controls. The lower concentrations of medium-chain fatty acid-derived acylglycines (hexanoylglycine and octanoylglycine) were not statistically significant. Nonetheless, the kinetic data showed significantly slower synthesis rates for acetyglcyine, BCAA-derived acylglycines, and medium-chain fatty acid-derived acylglycines in participants with obesity than healthy weight controls. Post-bariatric surgery, the urine concentrations and synthesis rates of acylglycines derived from acetylCoA, BCAAs, and medium-chain fatty acids increased significantly.

To examine whether obesity-associated glycine deficiency impairs the clearance of metabolites from exogenous sources, we measured the urine concentration and synthesis rate of benzoylglycine, also known as hippuric acid. Benzoic acid, a carboxylic acid used as a food preservative and a by-product of gut microbiome metabolism, is conjugated with glycine and excreted as bezoylglycine (9, 13). This pathway is linked to glycine homeostasis as glycine used to form benzoylglycine cannot be recycled and could drain the glycine pool (28). We found lower urine concentration and synthesis of benzoylglycine in individuals with obesity compared to controls. When glycine availability improved after bariatric surgery, synthesis and urine excretion of benzoylglycine increased. Regression analysis further revealed that a 16% variance in benzoic acid elimination can be attributed to glycine availability. These findings are consistent with our hypothesis that obesity-associated glycine deficiency impairs the glycine conjugation pathway and compromises the elimination of endogenous and exogenous metabolites as acylglycines.

Our findings have several potential clinical implications. The accumulation of incompletely oxidized substrates in metabolically active organs such as the skeletal muscle, heart, and liver can lead to mitochondrial stress and organ dysfunction (11, 12). Therefore, methods that enhance the elimination of excess metabolites via glycine conjugation could be developed to treat various obesity-related metabolic complications. Supplementation with dietary glycine is an antidote for humans with isovaleryl-CoA dehydrase deficiency by increasing organic acid excretion as acylglycine (29). Since the glycine conjugation reaction occurs primarily in the liver, patients with metabolic liver disease may benefit the most from such treatment. Oral glycine supplementation in high-fat diet Zucker-Fatty Rats enhanced urine acylglycine excretion, reduced serum triglycerides, and decreased hepatic short- and medium-chain acylCoAs. However, the actual metabolic benefit at the organ level remains to be determined (23). Another study also showed that glycine supplementation in animals with non-alcoholic hepatic liver disease lowered serum triglycerides, hepatic steatosis, and inflammation (30). However, whether these improvements were attributable to the glycine conjugation pathway or other mechanisms was unclear. Further studies in humans are needed to examine the effectiveness of glycine supplements to improve glycine conjugation as a treatment for specific obesity-related metabolic complications. The impaired elimination of benzoic acid in patients with obesity indicates that the detoxification of xenobiotic metabolites via glycine conjugation is compromised. Humans in the industrialized world are exposed to various chemicals in the environment, food, and consumer products. Some of these molecules are regarded as endocrine-disrupting chemicals and may contribute to various endocrine disorders (31). Thus, individuals with class III obesity may be more susceptible to various environmental toxins due to glycine deficiency, but this hypothesis needs to be tested in future studies.

Our study has several limitations. We reported the effect of glycine deficiency on the glycine conjugation pathway and discussed its metabolic implications when this detoxification system is compromised. However, glycine plays a central role in various human metabolic reactions. Our understanding of the metabolic consequence of glycine deficiency is incomplete without examining its impact on other physiologically important pathways, such as 1-carbon cycle metabolism, glutathione biosynthesis, glycine-mediated signaling pathways, and gut microbiome. For example, higher glycine availability could modulate the gut microbiome population, although the causal relationship between gut microbiome and disease pathogenesis remains complex (30). We could not measure the urine concentrations and synthesis rates of long-chain fatty acids-derived acylglycines due to technical difficulties in preparing its internal standards. However, the glycine conjugation pathway may also have greater selectivity for acylCoAs of short and medium-chain lengths. We studied and reported the improvement in the glycine conjugation pathway by correcting glycine deficiency in individuals who underwent bariatric surgery. However, bariatric surgery also changes body weight, body composition, diet, and physical activity levels. It is unclear whether these contemporaneous changes confound our measured outcomes and should be controlled for in future studies.

## 6 Conclusion

Obesity-associated glycine deficiency impairs the human body’s ability to eliminate endogenous and exogenous metabolites via the glycine conjugation pathway. However, this impairment can be ameliorated by correcting glycine deficiency with weight loss secondary to bariatric surgery.

## Data availability statement

The raw data supporting the conclusions of this article will be made available by the authors, without undue reservation.

## Ethics statement

The studies involving humans were approved by SingHealth Centralized Institutional Review Board. The studies were conducted in accordance with the local legislation and institutional requirements. The participants provided their written informed consent to participate in this study.

## Author contributions

HT: Conceptualization, Data curation, Formal analysis, Funding acquisition, Investigation, Methodology, Project administration, Resources, Visualization, Writing – original draft, Writing – review & editing. JH: Conceptualization, Formal Analysis, Methodology, Writing – original draft. ET: Supervision, Writing – review & editing. SC: Writing – review & editing, Methodology. JK: Writing – review & editing. FJ: Methodology, Writing – review & editing, Conceptualization, Supervision.
